# Cobimetinib and trametinib inhibit platelet MEK but do not cause platelet dysfunction

**DOI:** 10.1080/09537104.2018.1514107

**Published:** 2018-09-25

**Authors:** Amanda J. Unsworth, Alexander P. Bye, Neline Kriek, Tanya Sage, Ashley A. Osborne, Dillon Donaghy, Jonathan M. Gibbins

**Affiliations:** 1 Institute for Cardiovascular and Metabolic Research, University of Reading, School of Biological Sciences, Reading, UK; 2 Department of Microbiology Immunology and Pathology, Colorado State University, Fort Collins, CO, USA

**Keywords:** ERK, MEK, platelets, thrombosis

## Abstract

The MEK inhibitors cobimetinib and trametinib are used in combination with BRAF inhibitors to treat metastatic melanoma but increase rates of hemorrhage relative to BRAF inhibitors alone. Platelets express several members of the MAPK signalling cascade including MEK1 and MEK2 and ERK1 and ERK2 but their role in platelet function and haemostasis is ambiguous as previous reports have been contradictory. It is therefore unclear if MEK inhibitors might be causing platelet dysfunction and contributing to increased hemorrhage. In the present study we performed pharmacological characterisation of cobimetinib and trametinib *in vitro* to investigate potential for MEK inhibitors to cause platelet dysfunction.

We report that whilst both cobimetinib and trametinib are potent inhibitors of platelet MEK activity, treatment with trametinib did not alter platelet function. Treatment with cobimetinib results in inhibition of platelet aggregation, integrin activation, alpha-granule secretion and adhesion but only at suprapharmacological concentrations. We identified that the inhibitory effects of high concentrations of cobimetinib are associated with off-target inhibition on Akt and PKC. Neither inhibitor caused any alteration in thrombus formation on collagen under flow conditions in vitro.

Our findings demonstrate that platelets are able to function normally when MEK activity is fully inhibited, indicating MEK activity is dispensable for normal platelet function. We conclude that the MEK inhibitors cobimetinib and trametinib do not induce platelet dysfunction and are therefore unlikely to contribute to increased incidence of bleeding reported during MEK inhibitor therapy.

## Introduction

Protein kinases mediate many critical signalling events in platelet activation and enable them to fulfil their role in primary haemostasis. Although platelet kinases have not yet been successfully targeted for antiplatelet therapy, many kinase inhibitors are now being used to treat other diseases, including cancers. Some cancer therapies target kinases that are also present in the platelet and can cause platelet dysfunction and increase risk of bleeding events. Dasatinib (Src), ibrutinib (Btk) and imatinib (Bcr-Abl) all cause platelet dysfunction via inhibition of platelet kinases, while drugs like idelalisib (PI3K, P110δ) inhibit platelet kinases without increasing bleeding risk. This necessitates strategies to manage bleeding risk, such as contraindication with antiplatelet medication or cessation of therapy prior to surgical procedures.

The MEK inhibitors trametinib and cobimetinib were approved for the treatment of metastatic melanoma in 2013 and 2015, respectively, and target MAPK signalling which is upregulated in several forms of cancer. Increased rates of haemorrhage with MEK inhibitor plus BRAF inhibitor dabrafinib compared to dabrafinib plus placebo have been recorded in multiple clinical trials evaluating trametinib [1,2] or cobimetinib. In the majority of cases investigators believed these events to be non-treatment related, yet one case of intracranial haemorrhage was attributed to therapy and not tumour cells [3]. Given the ambiguity of the role of MEK in platelet function and primary haemostasis, it is not clear if increased rates of bleeding might be exacerbated by drug-induced platelet dysfunction.

Cobimetinib and trametinib are highly selective, allosteric, non-ATP competitive MEK 1 and 2 inhibitors [4,5]. Cobimetinib binds to phosphorylated MEK (with a 100x greater potency for MEK1 over MEK2) whilst trametinib binds to non-phosphorylated MEK1 and 2 with similar potencies, though both inhibit downstream ERK signalling. Both cobimetinib and trametinib are used to inhibit *BRAFV600E/K*-mediated MEK1 activation, phospho-MEK1 activity, cellular phosphorylation of ERK, and cellular proliferation. Both inhibitors display anticancer activity against metastatic melanoma carrying the *BRAF* V600E/K mutation. Platelets are known to express MEK1 and MEK2 [6] and their downstream effectors ERK1, and ERK2 [7,8] as well as p38^MAPK^ (α and β2 isoforms and low levels of the γ and δ isoforms) [9] and JNK MAPKs members, yet the role of this pathway in platelet function has remained controversial. In platelets, ERK2 activation is thought to be mediated by MEK1/2 and seems to be dependent on protein kinase C (PKC) [10].

Studies using pharmacological MAPK inhibitors have reported the involvement of MAPK signalling pathways in vWF/GPIb-IX dependent platelet aggregation [11,12], and the regulation of platelet functional responses following stimulation by other platelet agonists, including regulation of integrin activity [12,13] and adhesion, spreading and thrombus formation on collagen [14]. Injection with a MEK inhibitor also inhibited thrombus formation *in vivo* and caused thrombus destabilisation when administered following injury of a blood vessel in mouse [15]. However, other studies have found that pharmacological inhibition of MEK does not inhibit platelet aggregation [16] or release of secondary mediators that support platelet function [17] and have shown that commonly used MEK inhibitors such as PD0325901 and U0126 inhibit other platelet kinases, possibly explaining their reported effects on platelet function.

In the present study, we performed thorough pharmacological characterisation of trametinib and cobimetinib in the context of platelet signalling and function to determine whether MEK inhibition or indeed off-target inhibition of other platelet signalling processes by the MEK inhibitors could cause platelet dysfunction *in vitro*.

## Methods

### Materials

Cobimetinib and Trametinib was obtained from SelleckChem (Stratech, Suffolk, UK). Type I Collagen was obtained from Nycomed (Munich, Germany) and collagen-related peptide (CRP-XL) from Professor Richard Farndale (University of Cambridge, Cambridge, UK). ADP, epinephrine, PMA, Thrombin and U46619 were from Sigma (Gillingham, UK), PAR1 activating peptide TRAP-6 (SFLLRN) was from Bachem (Switzerland). Phospho-PKC substrate antibody, Phospho-p44/42MAPK (Erk1/2) (Thr202/Tyr204), Phospho-Akt S473 and Phospho-Akt T308 was from New England Biolabs (Hitchin, UK). Phospho Src Y418 was from Merck Millipore, Hertfordshire, UK. β-Actin antibody was from Abcam (Cambridge, UK). FITC-conjugated anti-fibrinogen antibody was from Agilent Technologies LDA UK Limited, (Cheadle, UK) and PE-Cy5 anti- CD62P was from BD Biosciences (Oxford, UK). Half area 96-well plates were from Greiner BioOne (Stonehouse, UK) and DiOC6 was from Thermo Fisher Scientific (Dartford, UK). For antibody catalogue numbers please refer to major resources table in the supplemental material.

### Platelet preparation

Blood samples were obtained from healthy, aspirin free, consenting volunteers, using procedures approved by the University of Reading Research Ethics Committee. Blood was collected into a syringe containing 3.8% (w/v) sodium citrate and acid citrate dextrose (ACD) was added prior to preparation of platelet-rich plasma (PRP). Washed platelets were prepared by first isolating the PRP following centrifugation at 100 g for 20 minutes at room temperature, and centrifugation once at 350 g for 20 minutes to pellet the platelets. Platelets were resuspended at at 4 × 10^8^ cells/ml in modified Tyrode’s-HEPES buffer, (134 mM NaCl, 0.34 mM Na2HPO4, 2.9 mM KCl, 12 mM NaHCO3, 20 mM N-2-hydroxyethylpiperazine-N′-2ethanesulfonic acid, 5 mM glucose and 1 mM MgCl2, pH 7.3) and kept at 30°C before being used immediately [18].

### Aggregometry

For measurement of plate based aggregation in washed platelets: half area 96-well plates (Greiner) containing platelet agonists at a range of concentrations: ADP, CRP-XL, U46619, collagen, TRAP-6 and PMA and washed platelets (4 x 10^8^/ml) were (loaded onto plates) and shaken at 1,200rpm for 5 minutes at 37°C using a plate shaker (Quantifoil Instruments) as described by Lordkipanidzé et al [19] and absorption of 405 nm light measured using a Multiskan Ascent 354 Microplate Reader (LabSystems). Traditional light transmission based real time aggregometry was measured using washed platelets (4 x 10^8^/ml) under stirring conditions (1200 rpm) at 37°C for 5 minutes in an AggRAM aggregometer (Helena Biosciences, Europe).

### FACS measurement of fibrinogen binding, P-selectin exposure and Annexin V binding

Measurements of fibrinogen binding, P-selectin exposure and Annexin V binding were performed using washed platelets pretreated with inhibitors as described in figure legends and stimulated with CRP-XL (1 µg/ml) or CRP-XL (1 µg/ml) and TRAP-6 (1 µM) in the presence of FITC-conjugated polyclonal rabbit anti-fibrinogen antibody, PE-Cy5-conjugated mouse anti CD62P antibody or FITC-conjugated Annexin V, and then incubated for 20 minutes in the dark. Platelets were then fixed by addition of filtered formyl saline (0.2% formaldehyde in 0.15 M NaCl) and median fluorescence intensities were measured for 5000 events in the platelet gate (determined by forward and side scatter profiles) per sample on an Accuri C6 Flow Cytometer (BD Biosciences, UK) using CFlow Sampler software.

### Thrombus formation under flow

Thrombus formation was performed using blood from healthy donors using microfluidic flow chips (Vena8, Cellix Ltd, Dublin, Ireland) coated with 100 µg/ml type I collagen and measured in real time for 9 minutes, at a perfusion shear rate of 1000 s^−1^ following incubation of blood with cobimetinib, trametinib or vehicle for 10 minutes.

### Protein phosphorylation studies, immunoblotting

Washed platelets at 4 × 10^8^ cells/ml were incubated with cobimetinib or trametinib at concentrations specified in individual figures or vehicle for 10 minutes at 37°C prior to addition of 1 µg/ml CRP-XL or 1 µM TRAP-6. Samples were incubated for 90 secs and 60 seconds respectively with stirring at 37°C using an aggregometer (Helena) prior to addition of reducing Laemmli sample treatment buffer. Immunoblotting was performed using standard techniques as described previously [20]. Levels of phosphorylated proteins were detected using phospho-specific primary antibodies and fluorophore conjugated secondary antibodies and visualised using a Typhoon Trio fluorescence imager and Image Quant software (GE Healthcare). Band intensities were quantified and levels of the immunoprecipitated protein were used to control for protein loading using Image Quant software.

### Platelet adhesion

Washed platelets at 2 × 10^7^ cell/ml were exposed to collagen- (100 µg/ml), fibrinogen- and von-Willebrand factor-coated 96-well assay plates and allowed to adhere for 45 minutes at 37°C. Non-adherent platelets were removed by washing in PBS before fixing with 10% formyl saline for 10 minutes. The wells were then washed and labelled with DiOC6. Fluorescence images of adherent platelets were captured with the 20 x objective lens of an ImageXpress Nano high content imaging system and counted using CellReporterXpress software (Molecular Devices, Winnersh, UK).

### Statistical methods

Statistical testing described in figure legends and the results section were performed using GraphPad Prism Software (Graphpad, La Jolla, CA). Where data are normalised, this was done to control for unwanted sources of variation and statistical analysis was performed prior to normalisation. P ≤ 0.05 was considered statistically significant. Unless stated otherwise, values are expressed as mean ±SEM.

## Results

### MEK inhibitors induce platelet dysfunction only at suprapharmacological concentrations

**Figure 1.**
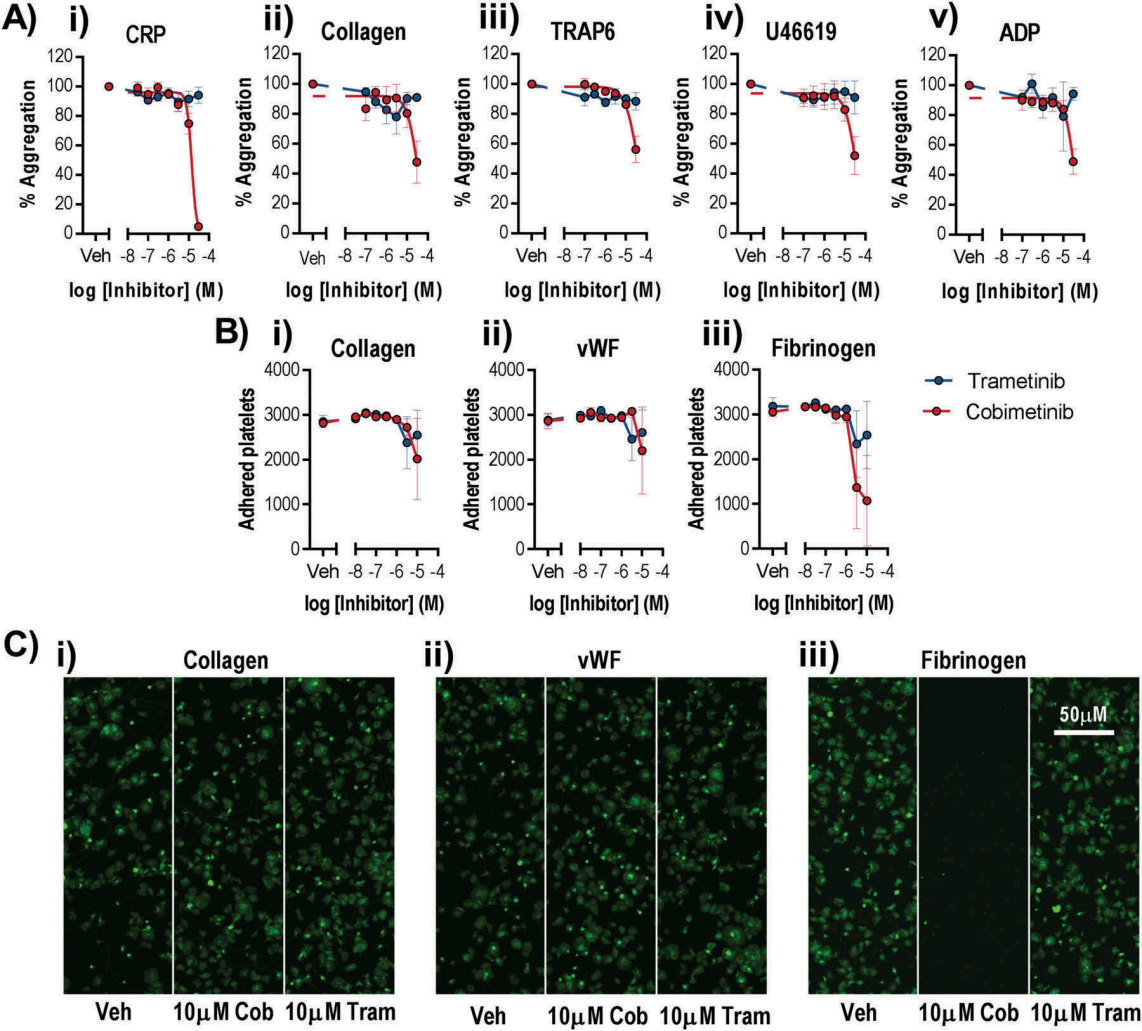
Only suprapharmacological concentrations of MEK inhibitors cause dysfunctional platelet aggregation and adhesion. Washed human platelets were pre-treated with increasing concentrations of Cobimetinib (red) or Trametinib (blue) (0.1–100 µM) for 10 minutes prior to a) stimulation with either i) CRP (1 µg/ml), ii) collagen (1 µg/ml), iii) TRAP-6 (10 µM), iv) U46619 (1 µM) or v) ADP (10 µM) and the extent of platelet aggregation was monitored after 5 minutes of shaking using an optical light transmission plate based aggregometry assay, and concentration response curves b) and representative images c) of platelets adhered to i) collagen (10 µg/ml), ii) vWF (10 µg/ml) or iii) fibrinogen (100 µg/ml) coated wells of microtitre plates for 1 hour at room temperature, data expressed as number of adhered platelets (platelet count). Results are mean + S.E.M. for n ≥ 3, * indicates p ≤ 0.05 in comparison to vehicle controls.

To investigate whether MEK inhibitor-induced platelet dysfunction might contribute to the increased incidence of bleeding observed during MEK inhibitor therapy, we first investigated platelet aggregation using both a high-throughput microtitre plate-based assay (Figure 1a). Washed platelets were treated with a range of MEK inhibitor concentrations and then stimulated with 1 µg/ml CRP-XL, 1 µg/ml collagen, 10 µM TRAP-6 (SFLLRN), 1 µM U46619 or 10 µM ADP. Trametinib did not inhibit platelet aggregation evoked by any of the agonists even at the highest concentration tested (30 µM). In contrast cobimetinib inhibited aggregation evoked by all of the agonists, although with low potency and failed to totally ablate aggregation evoked by any of the agonists except CRP (log IC_50_ = −4.9 M ± 0.02). Similar results were also observed following stimulation by lower concentrations of platelet agonists (0.1 and 0.3 µg/ml CRP-XL and 0.1, 1 µM TRAP-6) indicating MEK inhibition does not just occur at low concentrations of platelet agonists (Supplementary Figure 1). Further investigation of the effect of the MEK inhibitors on the profile of platelet aggregation using real-time traditional light transmission aggregometry identified no inhibition of the kinetics or extent of platelet aggregation to either 1 µg/ml CRP-XL or 1 µM TRAP-6 by either cobimetinib or trametinib even at the highest concentrations tested (10 µM) (Supplementary Figure 1). The effects of the MEK inhibitors on adhesion of platelets to collagen, vWF or fibrinogen coated surfaces (Figure 1b-c) was also determined using a microtitre plate-based method. We found that both trametinib and cobimetinib were weak inhibitors of adhesion to collagen and vWF but that cobimetinib inhibited adhesion to fibrinogen strongly in some experiments, but only at high concentrations (≥ 3 µM).

**Figure 2.**
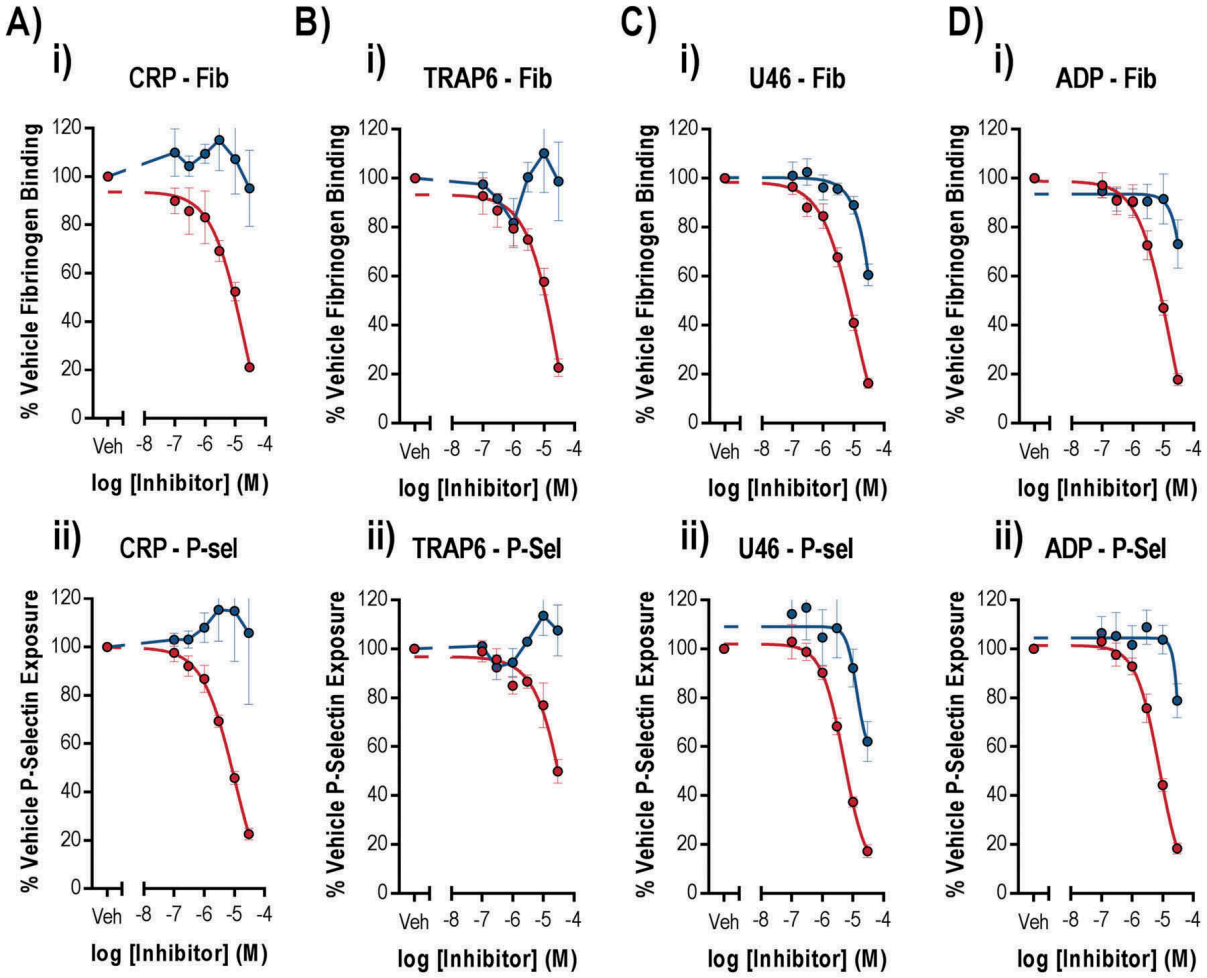
Only suprapharmacological concentrations of MEK inhibitors inhibit integrin α_IIb_β_3_ activation and alpha granule secretion. Washed human platelets were pre-treated with increasing concentrations of Cobimetinib (red) or Trametinib (blue) (0.1–30 µM) for 10 minutes prior to stimulation with (a) CRP (1 µg/ml), (b) TRAP-6 (10 µM), (c) U46619 (1 µM) or (d) ADP (10 µM) for 20 minutes before cells were fixed and (i) fibrinogen binding and (ii) P-selectin exposure were measured by flow cytometry. Results are mean + S.E.M. for n ≥ 3, * indicates p ≤ 0.05 in comparison to vehicle controls.

The effects of cobimetinib and trametinib on integrin α_IIb_β_3_ activation and α granule secretion were determined using flow cytometry to measure fibrinogen binding and P-selectin exposure, as these processes are critical for platelet function (Figure 2). As observed for aggregation, we found that cobimetinib inhibited CRP-evoked fibrinogen binding (logIC_50_ = −5.2 M ± 0.17) and P-selectin exposure (logIC_50_ = −5.2 M ± 0.01) but that trametinib showed no inhibitory effect (Figure 2a). Similarly, activation by TRAP-6 was inhibited partially by cobimetinib, although not potently enough to enable estimation of IC_50_ values, and was not inhibited by trametinib (Figure 2b). Cobimetinib was also found to inhibit U46619-evoked integrin α_IIb_β_3_ activation (logIC_50_ = −4.9 M ± 0.09) and P-selectin exposure (logIC_50_ = −5.2 M ± 0.04) while trametinib only partially inhibited these responses even at the highest concentration tested (30 µM) (Figure 2c). Similar to the results seen with U46619, cobimetinib inhibited ADP-evoked integrin α_IIb_β_3_ activation (logIC_50_ = −5.2 M ± 0.25) and P-selectin exposure (logIC_50_ = −5.1 M ± 0.04) while trametinib only partially inhibited at 30 μM (Figure 2d).

Exposure of phosphatidylserine (PS) is an key step in the formation of procoagulant platelets which supports the processes of coagulation that enable stable thrombus formation. The effects of cobimetinib and trametinib on Annexin V binding were determined using flow cytometry (Supplementary Figure 2a). We found that neither cobimetinib or trametinib were capable of stimulating PS exposure in the absence of platelet stimulation, indicating neither MEK inhibitor elicits procoagulant platelet formation or initiates platelet apoptosis. As observed for other platelet functional responses, we found that cobimetinib inhibited Annexin binding (logIC_50_ = −5.1 M ± 0.76) following platelet stimulation with a combination of CRP-XL (1μg/ml) and TRAP (10 μM), but that trametinib showed no inhibitory effect (Supplementary Figure 2b).

**Figure 3.**
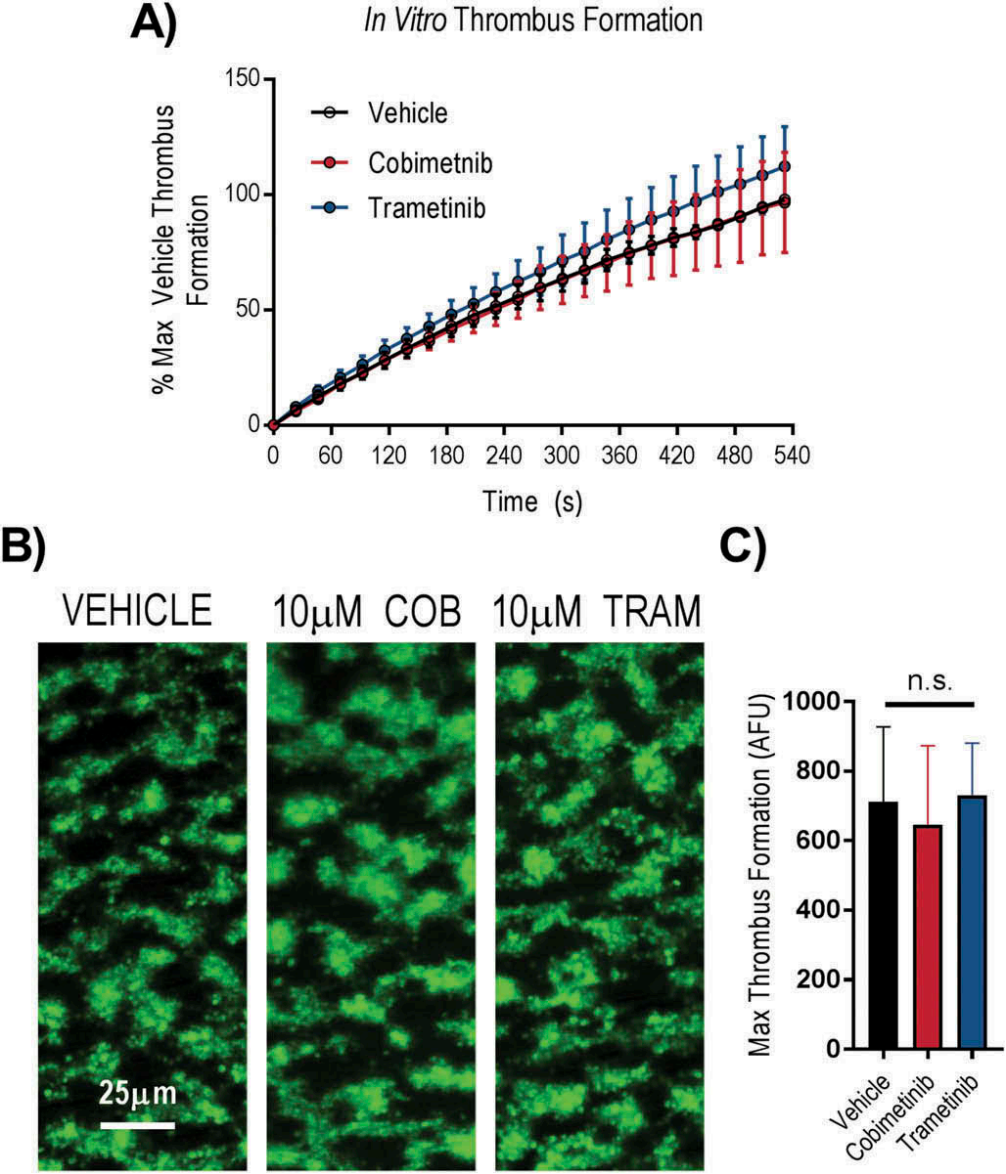
MEK inhibitors do not cause dysfunctional thrombus formation in whole blood. DiOC_6_ loaded human whole blood was pre-treated with vehicle (black), 10 µM cobimetinib (red) or 10 µM trametinib (blue) for 10 minutes, before perfusion through collagen coated (100 µg/ml) Vena8Biochips at a shear rate of 1000s^−1^. (a) Real time thrombus formation was determined for 9 minutes by comparing fluorescence intensity in the vehicle and treated samples. Maximum fluorescence observed in the vehicle control was set at 100%. (b) Representative images taken at 9 minutes are shown. (c) Maximum thrombus formation determined by maximum fluorescence and expressed as arbitrary fluorescence units. Results are mean ±S.E.M. for n ≥ 3, * indicates p ≤ 0.05 in comparison to vehicle controls.

The ability of platelets to form stable aggregates under arterial shear is critical for normal haemostasis. We therefore investigated the effects of cobimetinib and trametinib in an in vitro assay of thrombus formation under arterial flow conditions using whole blood (Figure 3). We found that accumulation of platelets on collagen coated flow chambers, normalised to maximum vehicle-treated response to compensate for differences between donors, occurred at a similar rate following treatment with 10 µM cobimetinib or trametinib compared to vehicle-treated controls (Figure 3a). The morphology of thrombi formed in either MEK inhibitor-treated whole blood appeared identical to vehicle-treated control (Figure 3b) and quantitation of non-normalised platelet deposition onto collagen after 9 minutes was not significantly different to vehicle-treated controls following treatment with either cobimetinib or trametinib (10 μM) (p > 0.05, 1-way ANOVA with matched pairs and Dunnett’s multiple comparison test, Figure 3c).

### MEK is dispensable for normal platelet function and signalling

**Figure 4.**
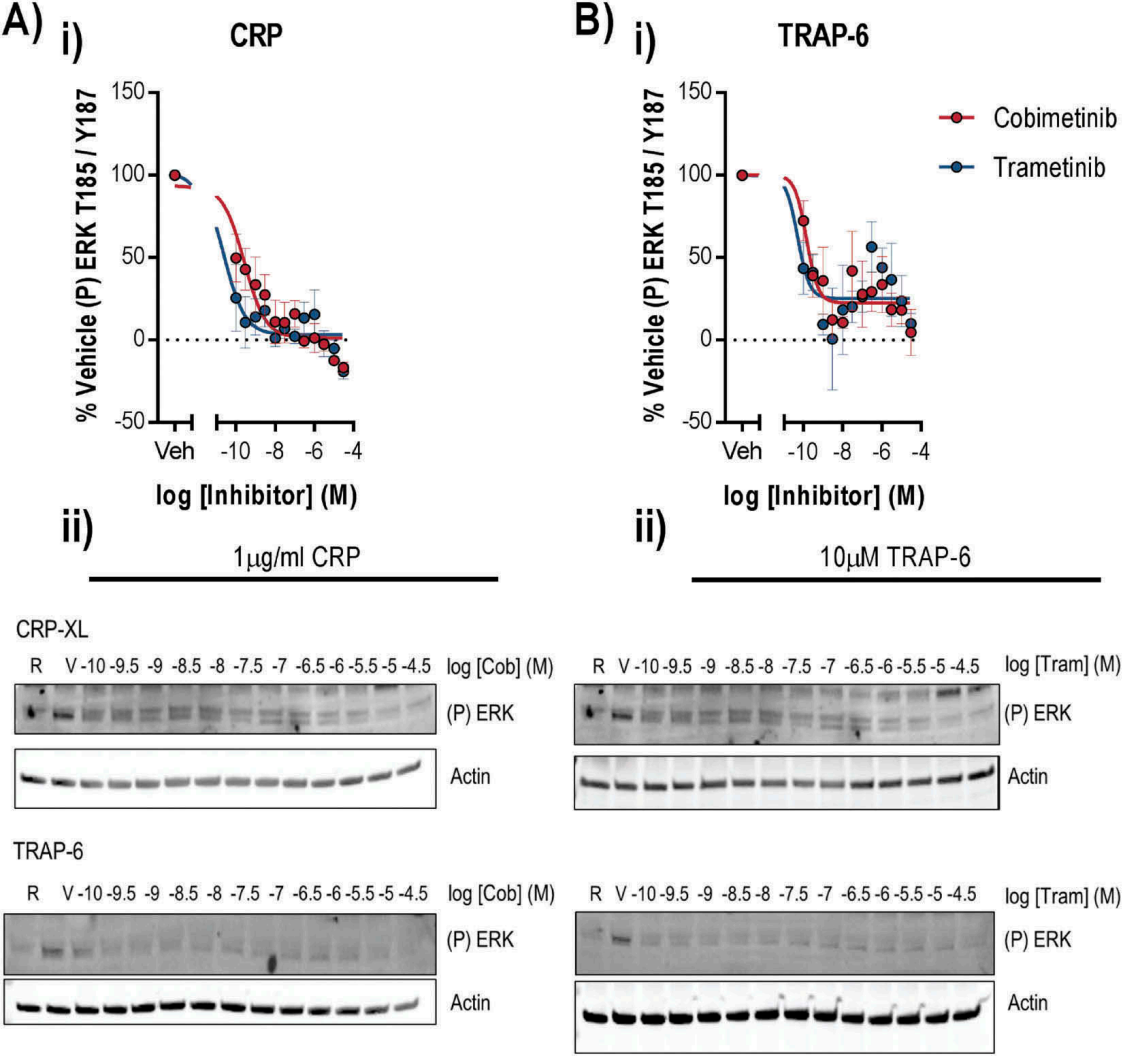
Cobimetinib and trametinib potently inhibit platelet MEK activity and phosphorylation of MEK substrate ERK1/2. Washed human platelets were pre-treated with increasing concentrations of cobimetinib or trametinib (0.1 nM-30 µM) for 10 minutes prior to stimulation with (a) CRP (1 µg/ml) or (b) TRAP-6 (10 μM) under aggregating conditions for 90 and 60 secs respectively before lysis in SDS Laemmli sample buffer, and western blotting for phosphorylation of ERK at T185/Y187. (i) Levels of total phosphorylation were quantified and expressed as a percentage of vehicle-treated stimulated controls and actin was used to confirm equal loading. (ii) Representative blots shown. Results are mean and ±S.E.M. for n ≥ 3, * p < 0.05, ** p < 0.01 relative to vehicle-treated controls.

Given the lack of potent antiplatelet effects following treatment of platelets with MEK inhibitors, we investigated platelet signalling events to clarify the role of MEK and other signalling kinases in platelet function. We found that stimulation of platelets with 1 µg/ml CRP or 10 µM TRAP-6 evoked robust ERK1/2 phosphorylation at threonine 185 and tyrosine 187 (Figure 4) which is mediated by MEK and is required for ERK1/2 kinase activity [21,22]. Treatment of platelets with cobimetinib potently inhibited ERK1/2 phosphorylation evoked by CRP (logIC_50_ = −9.5 M ± 0.53) and TRAP-6 (logIC_50_ = −9.5 M ± 0.47) (Figure 4a-b). Similar observations were also made following treatment with trametinib, with significant attenuation of ERK1/2 phosphorylation evoked by CRP (logIC_50_ = −10.9 M ± 0.43) and TRAP-6 (logIC_50_ = −9.6 M ± 0.44) (Figure 4a-b).

**Figure 5.**
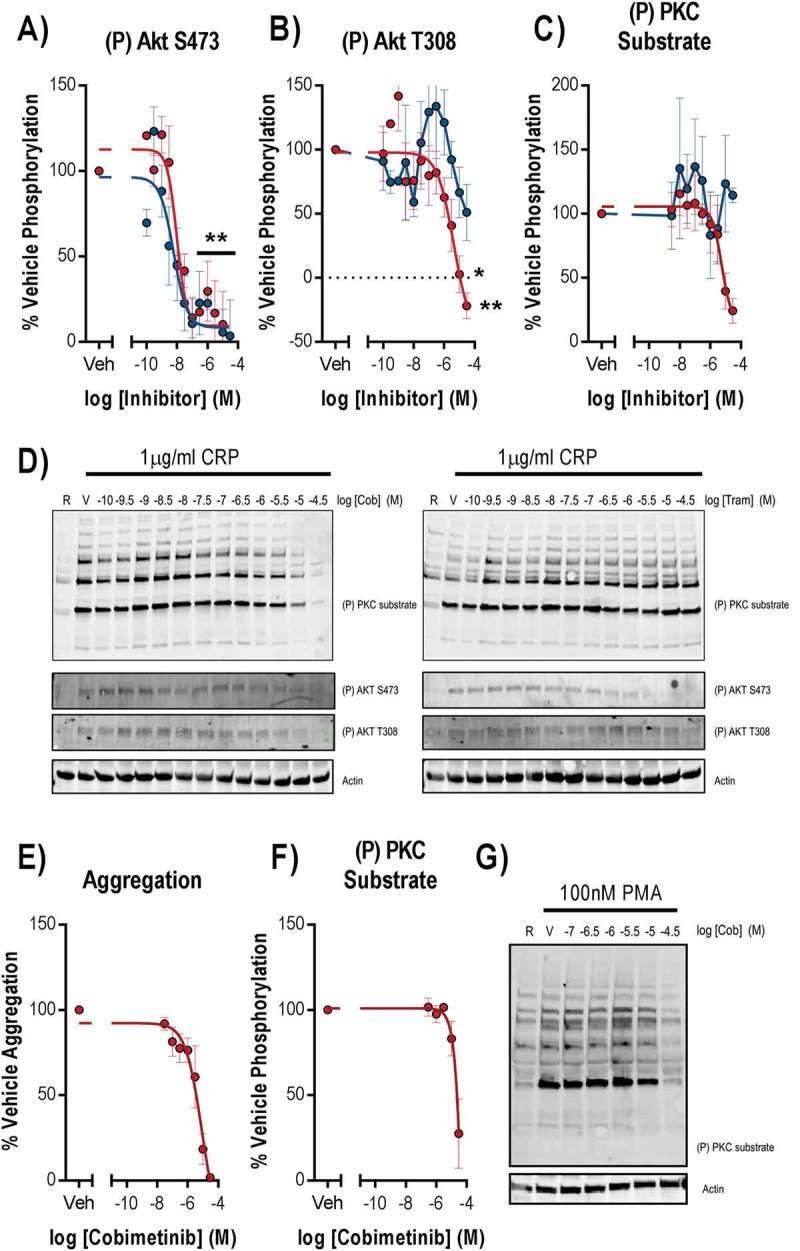
Inhibition of phosphorylation of the MEK substrate, ERK does not correlate with inhibition of platelet activation markers. Washed human platelets were pre-treated with increasing concentrations of cobimetinib or trametinib (0.1 nM-30 µM) for 10 minutes prior to stimulation with (a-d) CRP (1 µg/ml) for 90 seconds and (e) PMA (100 nM) for 10 minutes under aggregating conditions for 90 secs before lysis in SDS Laemmli sample buffer, and western blotting for phosphorylation of (a) Akt S473, (b) Akt T308 and (c) and (f) total PKC substrates using phospho-site specific antibodies. Levels of total phosphorylation were quantified and expressed as a percentage of vehicle-treated-stimulated controls and actin was used to confirm equal loading. (d) and (g) representative blots shown. (e) the extent of platelet aggregation was monitored after 5 minutes of shaking using an optical light transmission plate based aggregometry assay, and concentration response curves were plotted. Results are mean and ±S.E.M. for n ≥ 3, * p < 0.05, ** p < 0.01 relative to vehicle-treated controls.

As we observed cobimetinib and trametinib are both potent inhibitors of platelet MEK activity (nM) but weak inhibitors of platelet function (μM), we concluded that inhibition of MEK activity was unlikely to be the cause of the observed inhibition of platelet function. We therefore went on to investigate inhibition of other kinases that are important for platelet function that might cause platelet dysfunction observed at supraphysiological concentrations of cobimetinib (Figure 5).

mTOR and ILK mediate phosphorylation of Akt, a downstream effector of PI3K platelet signalling, at S473 and phosphorylation of this site was potently inhibited by both cobimetinib (logIC_50_ = −8.0 M ± 0.16) and trametinib (logIC_50_ = −8.2 M ± 0.28, Figure 5a-d). In contrast, the PDK1 phosphorylation site on Akt T308 was inhibited by cobimetinib but not by trametinib, and the inhibition of T308 observed with cobimetinib was with much lower potency (logIC_50_ = −5.4 M ± 0.65) than that observed for inhibition of Akt S473 (Figure 5b-d). We investigated PKC activity by measuring total phosphorylation of PKC substrates and found that cobimetinib inhibited PKC activation with low potency (logIC_50_ = −5.3 ± 0.28) and trametinib caused no inhibition of PKC activity (Figure 5c-d). Cobimetinib also caused inhibition of PMA induced platelet aggregation (Figure 5e) and PKC substrate phosphorylation (Figure 5f) strongly suggesting that cobimetinib may directly inhibit Ser/Thr kinases such as PKC and Akt and exert off-target effects on platelet function through this mechanism.

**Figure 6.**
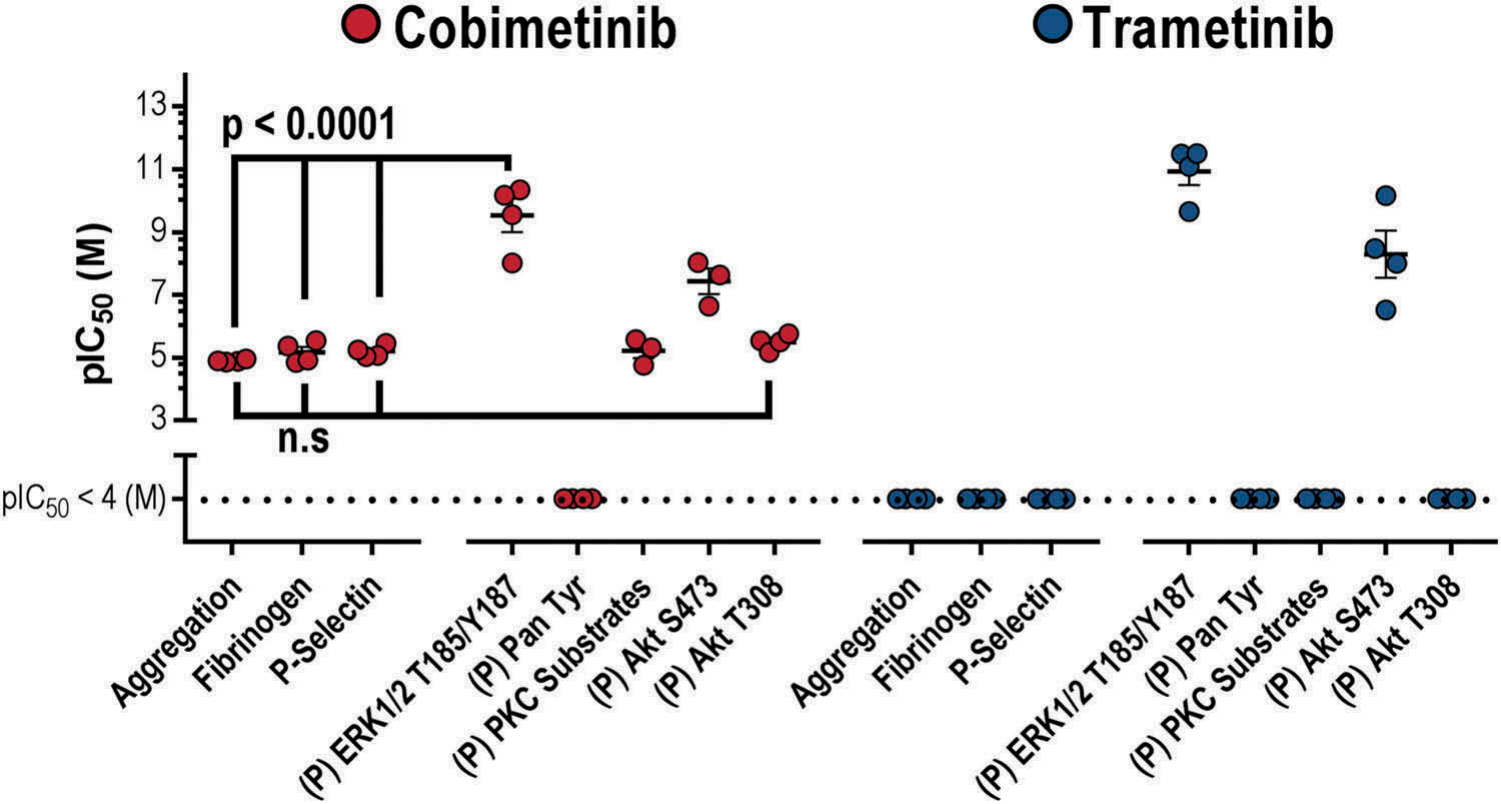
MEK activity is dispensable for platelet function.A scatter plot comparing the potencies (log IC_50_) of cobimetinib and trametinib in platelet function tests (aggregation, fibrinogen binding and p-selectin exposure) relative to inhibition of different platelet kinases.

In order to establish the how cobimetinib causes inhibition of platelet responses, we compared the potencies of the MEK inhibitors on platelet function with their effects on kinase activity (Figure 6). Although both kinases inhibit MEK and the kinases that mediate phosphorylation of Akt at S473, both trametinib and cobimetinib mediated these effects with a potency (IC_50_ nM) that is significantly greater than the potency with which they elicit inhibitory effects on platelet function (IC_50_ μM)). We therefore conclude that inhibitory effects on ERK1/2 T185/Y187 and Akt S473 are not related to the observed inhibitory effects on platelet function. In contrast, cobimetinib also inhibited PKC substrate phosphorylation and phosphorylation of Akt T308 with a potency that was not significantly different from that required to inhibit platelet function.

### Combined BRAF and MEK inhibitors do not impair platelet function

**Figure 7.**
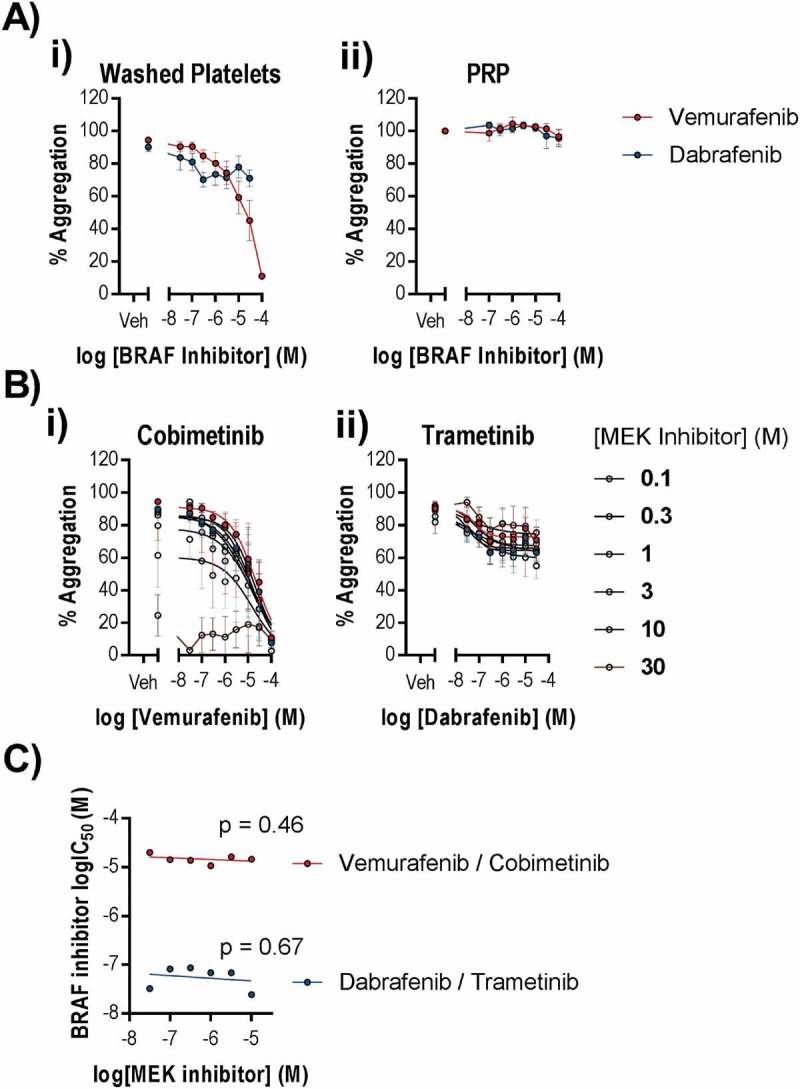
BRAF inhibitors do not potently inhibit platelet function alone or in combination with MEK inhibitors. Washed human platelets or PRP were pre-treated with increasing concentrations of Vemurafenib or Dabrafenib (0.1–100 µM) for 10 minutes in the (a) absence or (b) presence of increasing concentrations of b (i) cobimetinib or b (ii) trametinib respectively (0.1–30 µM), prior to stimulation by CRP (1 µg/ml) and the extent of platelet aggregation was monitored after 5 minutes of shaking using an optical light transmission plate based aggregometry assay. (c) comparison of the IC50’s for vemurafenib and dabrafenib in the presence of different concentrations of cobimetinib or trametinib respectively. Results are mean and ± S.E.M. for n ≥ 3, * p < 0.05, ** p < 0.01 relative to vehicle-treated controls.

MEK inhibitors are administered in combination with BRAF inhibitors to improve progression free survival. Although platelets express BRAF, a role for BRAF in normal platelet function has not been reported. However, given that cobimetinib and trametinib are administered with the BRAF inhibitors vemurafenib and dabrafenib, respectively, it was important to establish the effects of these drugs alone and in their therapeutic combinations to determine whether BRAF inhibitors cause platelet-dysfunction that could contribute to bleeding events in patients. We found that vemurafenib inhibited CRP-evoked platelet aggregation (logIC_50_ = 4.6 ± 0.61 (M), Figure 7a(i)) but not aggregation evoked by TRAP-6 (supplementary Figure 3). Dabrafenib did not inhibit aggregation evoked by CRP (Figure 7a(i)) or TRAP-6 (supplementary Figure 3). Vemurafenib is reported to have off target effects on the tyrosine kinase Src, which is critical for normal platelet function. In support of this, phosphorylation of Src at Y418 was reduced in CRP-stimulated platelets following treatment with vemurafenib at a similar potency (logIC_50_ = −4.9 ± 0.09 (M)) to the observed inhibition of platelet function (supplementary Figure 1). Although vemurafenib reaches therapeutic concentrations of up to 100 µM, it is highly bound by plasma proteins (approximately 99% plasma bound). In order to establish the effect of BRAF inhibitors on platelet function, we tested the potency of the BRAF inhibitors at inhibiting CRP-evoked aggregation of PRP and found that neither drug caused inhibition of aggregation in PRP even at 100 µM (Figure 7a(ii)). We tested for potential synergy between BRAF and MEK inhibitors by titrating the inhibitors against each other and measuring CRP evoked aggregation (Figure 7b(i) and b(ii)). We found no enhancement of potency (IC_50_) when combining vemurafenib with cobimetinib or dabrafenib with trametinib (non-zero gradient linear regression, p > 0.05, Figure 7c).

## Discussion

The MEK inhibitors cobimetinib and trametinib are currently used in combination with BRAF inhibitors for treatment of metastatic melanoma carrying the *BRAF* V600E/K mutation [4,5]. Increased rates of haemorrhage with cobimetinib or trametinib plus BRAF inhibitor dabrafinib compared to dabrafinib plus placebo have been recorded in several clinical trials [1,2]. Whether these events are treatment related is currently unclear [3]. Platelet dysfunction has been implicated in increased bleeding associated with other kinase inhibitor cancer therapies such as ibrutinib [18,23–26] and dasatinib [27]. Platelets express several members of the MAPK signalling cascade including both MEK1 and MEK2 and their downstream effectors ERK1 and ERK2. However, the role of MEK signalling in the regulation of platelet function is unclear, as several contradictory reports have been published [10–13,15–17]. As a result, the role for MEK in the regulation of platelet function, and the potential for MEK inhibitors such as cobimetinib and trametinib to adversely affect platelet function have remained ambiguous.

We investigated whether MEK inhibitors caused platelet dysfunction to establish whether this might contribute to increased risk of haemorrhage observed during therapy with MEK inhibitors. We identified that whilst both MEK inhibitors were highly potent inhibitors of MEK activity in platelets (Figure 4), cobimetinib inhibited important markers of platelet function; aggregation, adhesion, integrin activation, alpha granule secretion, phosphatidylserine exposure and thrombus formation, with low potency and trametinib showed virtually no inhibitory effect at all. Our findings clearly demonstrate that platelets are able to function normally under conditions where MEK activity is fully inhibited. Previous studies have reported a role for MEK signalling in thromboxane A2 generation downstream of GPIb [11] and the ADP receptors P2Y1 and P2Y12 [28]. However, we observed no effect of either MEK inhibitor on adhesion and spreading on vWF, or thrombus formation under flow, a test which is highly dependent on both vWF and TxA_2_ generation in citrated blood. We also observed no effect that could be attributed to inhibition of MEK, on aggregation, integrin α_IIb_β_3_ activation or α-granule secretion evoked by ADP.

It is possible that differences in assay design could underpin differences between our study and others, but we sought primarily to investigate the physiologically relevant effects of MEK inhibition on platelet function and were unable to identify any. A study of the MEK inhibitor U0126 found that concentrations of U0126 that fully inhibited ERK1/2 phosphorylation did not inhibit aggregation evoked by a range of different agonists, whereas inhibition of aggregation at higher concentrations was due to activity against targets other than MEK [16]. Another study using the MEK inhibitor PD 98059 found that inhibition of ERK1/2 activation by MEK did not alter platelet responses to the physiological agonists collagen or thrombin [17]. We believe that the disparity between the concentrations (nM) of cobimetinib required to inhibit MEK activity and the concentrations (μM) required to inhibit platelet function are due to off-target effects of the drug. These are most likely on PKC activity, either directly or through one of the kinases that activate PKC via phosphorylation of key residues in their catalytic domain, such as PDK-1 [29]. In support of this we found that phosphorylation of the PDK-1 substrate Akt T308 was inhibited by cobimetinib but not trametinib at supraphysiological concentrations, and we also observed inhibition of PKC substrate phosphorylation and aggregation following stimulation by the PKC activator PMA (Figure 5). Trametinib has greater selectivity for MEK than cobimetinib and the only MEK-independent signalling event that we found to be inhibited by trametinib was phosphorylation of Akt S473, a PDK-2 and ILK substrate, trametinib otherwise appears to lack the off-target effects on either platelet function or PKC activity observed with cobimetinib.

Following oral administration, cobimetinib is reported to reach a plasma concentration of approximately 18 nM [30] while trametinib reaches approximately 9.7 nM [31]. Following treatment with similar concentrations of cobimetinib or trametinib *in vitro*, whilst ablation of CRP or TRAP-6 evoked phosphorylation of ERK1/2 T185/Y187 was observed, no alteration in platelet aggregation, integrin activation, alpha granule secretion or adhesion and spreading on collagen, fibrinogen or vWF was detected. Although the concentration-effect relationship observed in vitro may not directly translate into the effects observed in vivo. The window between the effects on platelet function and MEK inhibition was sufficiently large (approximately 1,000-fold) that acute drug induced platelet dysfunction during treatment with cobimetinib or trametinib can effectively be ruled out.

As a strategy to minimise drug resistance, increase cancer therapy efficacy and overcome the Raf paradox, MEK inhibitors are administered in combination with BRAF inhibitors and so we felt it was important to ensure that combination of MEK inhibitors with their corresponding BRAF inhibitors did not synergise to cause platelet inhibition within the therapeutic range. BRAF is reported to be expressed in platelets, but a role for BRAF in platelet function has not previously been reported. We found that vemurafenib but not dabrafenib inhibited platelet function to both CRP and TRAP-6 at high but not pharamcologically relevant inhibitor concentrations, most likely due to off-target inhibition of Src phosphorylation and activity.

In summary, we found that both MEK inhibitors inhibited ERK phosphorylation potently, without affecting platelet function. The high degree of potency and selectivity at nanomolar concentrations of the inhibitors cobimetinib and trametinib enabled us to state confidently that phosphorylation of ERK by MEK is not required for normal platelet aggregation, adhesion, alpha granule secretion or thrombus formation. While cobimetinib was able to inhibit several functional platelet responses at high (suprapharmacological) concentrations, these were found to be due to off-target inhibition of platelet downstream signalling events including inhibition of both Akt and PKC activity, likely caused by inhibition of PDK1.

Study of the role of MEK and ERK in platelets has been highly dependent on pharmacological inhibitors due to the lack of viability of KOs and lack of nucleated cell models that would permit use of siRNA or other knockdown techniques. However, trametinib in particular is highly specific for MEK over other platelet kinases and therefore might offer the clearest answer yet as the importance of MEK and its regulation of ERK1/2 in platelets. Our findings suggest that ERK is either able to perform platelet-specific functions in the absence of MEK activity or that the use of pharmacological inhibitors in previous studies has led to some misattribution of platelet signalling roles to MEK-mediated activation of ERK1/2. ERK signalling is, however, important in the megakaryocyte where ERK1/2 regulates expression of genes involved in development and thrombopoeisis [32]. Other transcription factors, such as members of the nuclear receptor family [33–43], NFƙB, IƙBK and IKKB [44–47], and STAT3 [48,49] have been found to exert non-genomic effects on platelet function; however, it is possible that the MEK ERK signalling pathway could lead to a dead end due to the anucleate nature of the platelet.
