# Supplementary material for: Cobimetinib and trametinib inhibit platelet MEK but do not cause platelet dysfunction
Source: Platelets. 2018 Sep 25;30(6):762–72. doi: 10.1080/09537104.2018.1514107 (PMC6594423; doi:10.1080/09537104.2018.1514107)
Supplement: Supplemental Material [file IPLT_A_1514107_SM5319.pdf]

Supplemental Figures

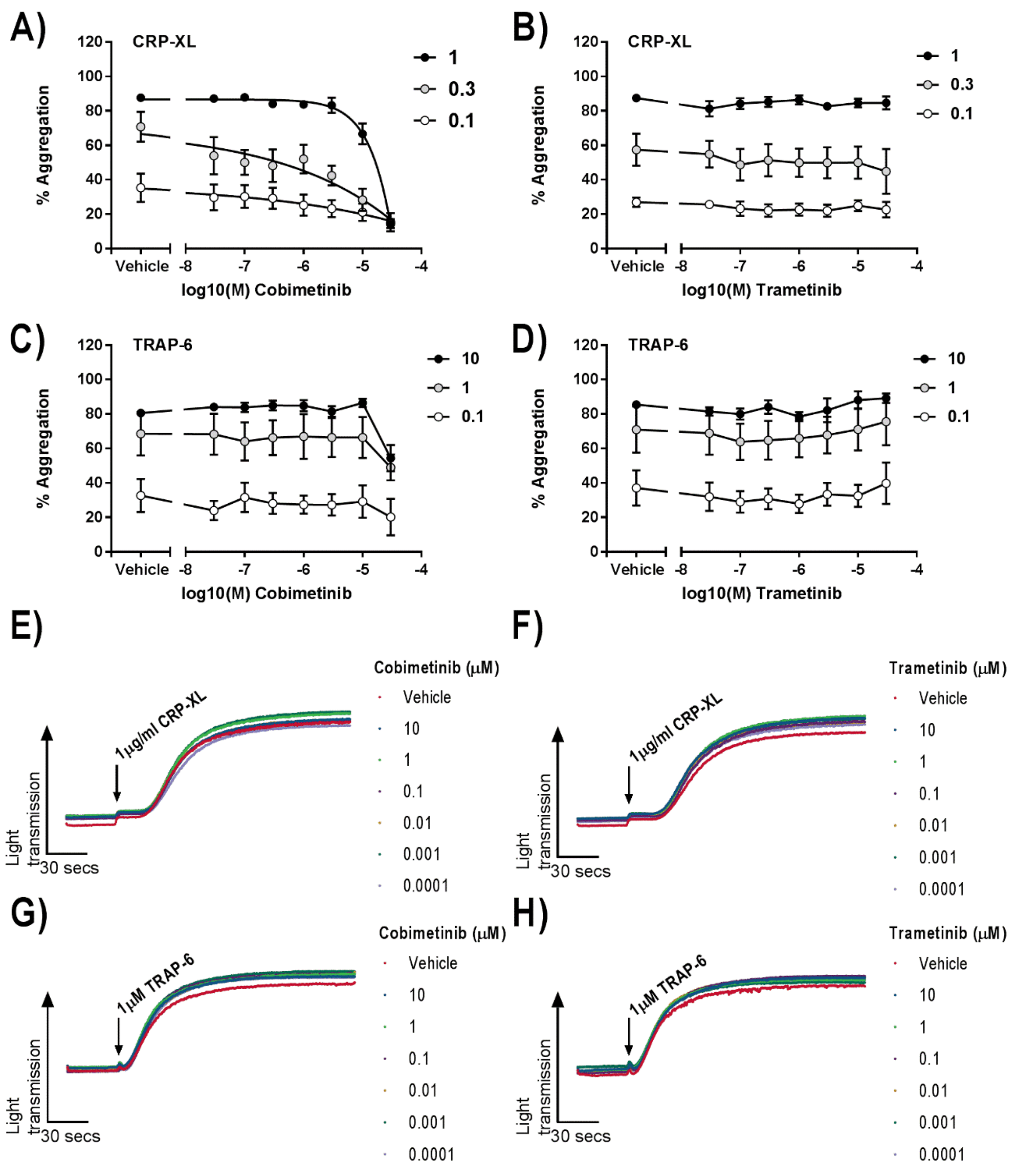

Supplementary Figure 1. MEK inhibitors do not inhibit platelet aggregation. Washed human platelets were pre-treated with increasing concentrations of Cobimetinib or Trametinib (0.0001- 30 μM) for 10 minutes prior to stimulation by A,B) CRP-XL (0.1, 0.3 or 1 μg/mL) or C,D) TRAP-6 (0.1, 1, 10 μM) and the extent of platelet aggregation was monitored after 5 minutes of shaking using an optical light transmission plate based aggregometry assay, results are mean and S.E.M, E, F) CRP (1μg/mL) or G,H) TRAP-6 (1 μM) and real time platelet aggregation was monitored for 5 minutes by measuring light transmission. Representative traces shown.

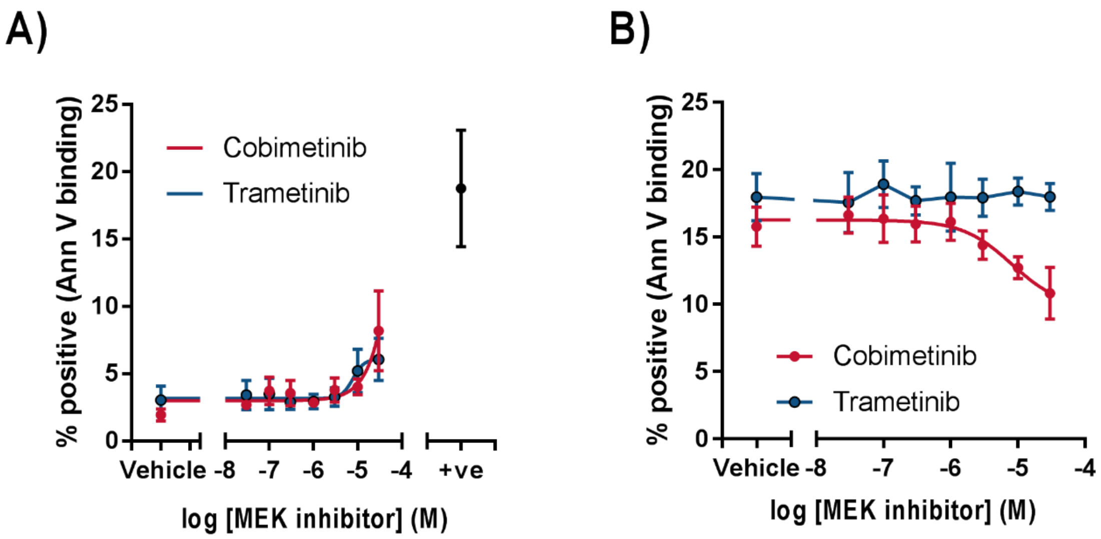

Supplementary Figure 2. The effect of MEK inhibition of platelet phosphatidylserine exposure. Washed human platelets were pre-treated with increasing concentrations of Cobimetinib or Trametinib (0.01- 30 µM) for 10 minutes and A) phosphatidylserine exposure was determined by measuring annexin V binding using flow cytometry. Platelets treated with CRP-XL (1 µg/mL) and TRAP-6 (10 µM) were included as a positive control. B) The effects of the MEK inhibitors on agonist-induced annexin V binding was measured following stimulation by a combination of CRP-XL (1 µg/mL) and TRAP-6 (10 µM). Data expressed as percentage of annexin positive cells. Results are mean ± S.E.M.

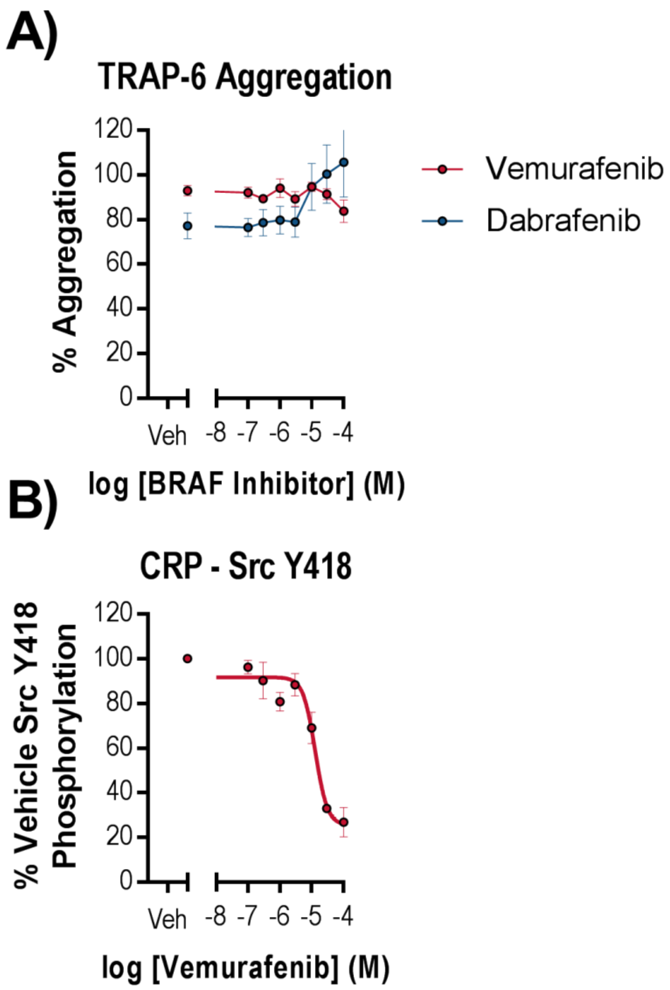

Supplementary Figure 3. BRAF inhibition does not induce platelet dysfunction. Washed human platelets were pre-treated with increasing concentrations of Vemurafenib or Dabrafenib (0.1-100 µM) for 10 minutes prior to stimulation by A) TRAP-6 (10 µM) and the extent of platelet aggregation was monitored after 5 minutes of shaking using an optical light transmission plate based aggregometry assay and B) CRP (1µg/ml for 90 seconds **and** SRC Y418 phosphorylation determined in samples lysed following stimulation with 1µg/mL CRP for 90 secs, following separation by SDS-PAGE gel electrophoresis and western blotting. Levels of total phosphorylation were quantified and expressed as a percentage of vehicle treated stimulated. Results are mean and s.e.m.
